# Universal opt-out screening for hepatitis C virus (HCV) within correctional facilities is an effective intervention to improve public health

**DOI:** 10.1108/IJPH-07-2016-0028

**Published:** 2017-09-11

**Authors:** Meghan D. Morris, Brandon Brown, Scott A. Allen

**Affiliations:** 1 Department of Epidemiology & Biostatistics, University of California, San Francisco, San Francisco, California, USA; 2 Center for Health Communities, University of California, Riverside, Riverside, California, USA; 3 School of Medicine, University of California, Riverside, Riverside, California, USA

**Keywords:** Criminal justice system, Public health, California, Epidemiology, HCV testing, Hepatitis C virus (HCV)

## Abstract

**Purpose:**

Worldwide efforts to identify individuals infected with the hepatitis C virus (HCV) focus almost exclusively on community healthcare systems, thereby failing to reach high-risk populations and those with poor access to primary care. In the USA, community-based HCV testing policies and guidelines overlook correctional facilities, where HCV rates are believed to be as high as 40 percent. This is a missed opportunity: more than ten million Americans move through correctional facilities each year. Herein, the purpose of this paper is to examine HCV testing practices in the US correctional system, California and describe how universal opt-out HCV testing could expand early HCV detection, improve public health in correctional facilities and communities, and prove cost-effective over time.

**Design/methodology/approach:**

A commentary on the value of standardizing screening programs across facilities by mandating all facilities (universal) to implement opt-out testing policies for all prisoners upon entry to the correctional facilities.

**Findings:**

Current variability in facility-level testing programs results in inconsistent testing levels across correctional facilities, and therefore makes estimating the actual number of HCV-infected adults in the USA difficult. The authors argue that universal opt-out testing policies ensure earlier diagnosis of HCV among a population most affected by the disease and is more cost-effective than selective testing policies.

**Originality/value:**

The commentary explores the current limitations of selective testing policies in correctional systems and provides recommendations and implications for public health and correctional organizations.

## Scope of the problem

Hepatitis C virus (HCV) infection is a global health issue of urgent importance. An estimated 170 million individuals are currently infected with HCV worldwide and result in 350,000-500,000 deaths a year. Globally, three to four million people are newly infected each year (Global Burden Of Hepatitis C Working Group, 2004; Mohd Hanafiah *et al.*, 2013). In middle- and high-income countries where the blood supply is screened, HCV is most often transmitted through contaminated needles and/or injection equipment. The prevalence of HCV is higher in correctional populations compared with the general population, with prevalence within correctional populations highest in Asia (40 percent), Australia (35 percent), and the USA (40 percent) (Larney *et al.*, 2013; Varan *et al.*, 2014). In the USA, HCV is the most common blood-borne viral infection with an estimated 2.5-3.2 million people in the general population infected with the chronic HCV (Beasley and Alter, 2010). And with more than ten million Americans move in and out of correctional facilities per year the correctional system offers an efficient, but often missed, opportunity for identification of people infected with HCV. Current estimates, based primarily on voluntary screening methods, suggest one in six prisoners in the US correctional system is infected with HCV (Varan *et al.*, 2014; Beckwith *et al.*, 2015).

Prison overcrowding, violence, and social isolation factor into prisoner initiation or continuation of behaviors that place them at risk for HCV infection (e.g. injection drug use) (Awofeso, 2010; Treloar *et al.*, 2015). Although forbidden in the correctional settings worldwide, illicit drug use often continues after imprisonment (22-50 percent of inmates have injected drugs) (He *et al.*, 2016). The resulting difficulty in obtaining sterile injection equipment promotes the sharing of infected equipment, which in turn facilitates the transmission of HCV. The estimated HCV incidence in prisons exceeds 30 per 100 persons per year (Teutsch *et al.*, 2010; Champion *et al.*, 2004; Christensen *et al.*, 2000). Due to low and varying HCV screening uptake in correctional settings, these numbers may underestimate the disease burden.

## Opportunities for expanded HCV screening in US correctional facilities

More precise estimates of the number of people infected with HCV cannot happen without improving our screening policies within correctional systems. HCV screening programs can fall into two main categories: opt-in or voluntary or opt-out or universal. Opt-in policies offer HCV testing only to individuals who specifically ask or who self-disclose as being a member of a risk-based group (e.g. individuals who disclose a history of injecting drugs). Opt-out or universal testing policies test everyone and individuals may choose whether to participate. Data from state corrections system directors on HCV screening procedures at state prison systems reported that only 17 states (34 percent) had at least one state-prison facility offering routine opt-out HCV screening, with eight (16 percent) having no screening program to capture HCV data in place (Beckwith *et al.*, 2015).

We argue for universal opt-out HCV screening of all persons upon entry into prison facilities. Universal opt-out HCV screening reflects the need for standardized protocols across facilities ensuring the same level of routine screening for all. When applied to other communicable diseases, opt-out screening procedures have resulted in earlier detection, reduced stigma, and increased number of screened individuals (Young *et al.*, 2009; Noland *et al.*, 2015), while allowing for infected individuals to decline. Due to considerable variability, universal opt-out HCV screening upon entry will improve the accuracy of HCV estimates, essential evidence for best resource allocation for prison health care and release programs. Compared to mandatory testing, which requires all individuals to have tested without the opportunity to decline and is a breach of human rights, opt-out testing programs within correctional faculties are typically accompanied by status disclosure (often in minutes thanks to rapid testing) and post-test counseling. Given the high prevalence of people who have injected drugs among correctional populations, a focused effort is needed to ensure these marginalized individuals are tested for HCV, engaged in medical care during incarceration, and linked to appropriate services upon release.

Given the number of people infected with HCV and the length of sentence stay (median stay in the USA was 65 months in 2009) (US Bureau of Justice Statistics, 2009), correctional facilities offer a unique opportunity for HCV screening to ensure more people know their status and earlier detection. Some estimates suggest that 40-70 percent of chronically infected individuals are unaware of their HCV positive status (Denniston *et al.*, 2012). Detecting HCV infection during a prison stay provides opportunities to ensure adequate care, education, and treatment assessment during incarceration and linkage-to-care post-release (Spaulding *et al.*, 2013). While routine opt-out testing has been shown to be feasible for other blood-borne pathogens within prison settings, the majority of prisons where some level of HCV screening exist, perform risk-based or selective screening, when testing for HCV (Rumble *et al.*, 2015; Beckwith *et al.*, 2015).

## Universal opt-out HCV screening can help inform resource allocation and benefits general public health

World Health Organization (2014) published the first set of recommendations for both screening and care of individuals with HCV, advocating that “HCV serology testing be offered to individuals who are part of a population with high HCV prevalence or who have a history of HCV risk exposure/behavior,” such as correctional populations. The Federal Bureau of Prisons (BOP) published guidelines for HCV screening in federal prisons in the USA. As of April 2016, the BOP recommends HCV infection testing for all sentenced prisoners at the prevention baseline visit who: disclose being a member of a high-risk group, have certain clinical conditions, and request testing (US Federal Bureau of Prisons, 2016). While these recommendations help reinforce the importance of devoting resources to specific groups, they fall short of enforcing the screening programs necessary to improve the health and safety of prison and general populations. More importantly, individual states are responsible for developing and implementing specific guidelines for HCV screening in state prisons. Individual state policies will affect roughly 80 percent of the US prison population.

Screening for HCV in prisons is a public safety precaution. HCV-infected individuals re-entering society may act as bridging populations, transmitting infections to the community via resumed injection drug use upon release. Each year in the USA, approximately one of every three people infected with HCV is part of the correctional system. After release, most (95 percent) return to their community-of-origin. Early detection and engagement in care can prevent prisoners from unwittingly transmitting HCV to others after re-entering the general community. Universal opt-out HCV screening at entry into prison may result in a dramatic reduction in the number of new HCV infections over the next 30 years, benefits able to extend beyond the prison environment to the general community.

This is a historic moment in the quest for HCV elimination thanks to the availability of novel, highly effective, therapy for HCV management. The recently available and highly effective oral direct-acting antivirals (DAAs) offer a cure for HCV-infected individuals. After treatment of one pill a day for 8-12 weeks, over 90 percent of patients reach undetectable viral levels and are “cured” of infection (Kohli *et al.*, 2014). The recent approval of a pan-genotypic single table regimen makes treating individuals independent of their HCV genotype a new reality (US Food and Drug Administration, 2016; Gilead, 2016). Compared to the old peg-IFN and ribavirin therapy, DAAs reduce treatment time and post-treatment follow-up, thus increasing the opportunity of completing treatment within prisons. The high costs of new DAAs are an important barrier for all HCV-positive individuals, even for those outside of the correctional system. A recent study of scope and cost of DAA treatment for HCV in state prisons reports that numerous departments of correction received smaller discounts on the prices of drugs than other state and national agencies. Smaller discounts resulted in state prisons paying between $50,000 and $92,000 USD per 12-week course of Ledipasvir/Sofosbuvir or Sofosbuvir (Beckman *et al.*, 2016). However, there is an opportunity for pooled procurement by state correctional systems to drive down the price of DAA medications. Australia and Canada are the example countries who have successfully negotiated down the price allowing for wide-scale treatment access and reduction in burden of HCV. This is another important reason to adopt opt-out HCV screening as improving the accuracy of HCV prevalence estimates in correctional systems can inform price negotiation strategies to reduce DAA treatment barriers.

In the following section, we provide a deeper look into the California State prison system, to illustrate the value of standardizing HCV screening programs across facilities by mandating all facilities (universal) to implement opt-out HCV screening policies for all prisoners upon entry to correctional facilities.

## California’s experience suggests that mandated universal (opt-out) testing in corrections is an essential and cost-effective policy

California state prison system is the largest prison system in the USA with 129,613 inmates in February 2017 (California Department of Corrections and Rehabilitation and Data Analysis Unit, February, 2017). Based on the current screening programs, it is estimated that one in three people imprisoned in California may be infected with HCV (33 percent), much higher than the number infected with HIV (2.5 percent) (Fox *et al.*, 2005). However, only a sub-sample of people in California prisons is tested, thereby leading to unreliable estimates of disease burden. Limited access to testing also means the majority is unaware of their HCV status. The passing of legislation (Assembly Bill 296, 2005) helped extend access to HCV testing within California state prisons by removing the $5 fee for HCV tests. That legislation also recommended that facilities implement voluntary (via request- or risk-based screening) HCV screening programs “only to the extent that funds for [HCV screening] are appropriated in the annual Budget Act” (2005). Under these current guidelines, how and when HCV testing is available to prisoners may differ from facility to facility, resulting in an unequal access across facilities. Additionally, voluntary, risk-based screening relies on the individuals to voluntarily disclose illegal and stigmatized behavior; something prisoners may be unwilling to reveal. Such a disclosure requirement is particularly ethically problematic in a system where medical staff are employed by the correctional system and may report behaviors. Updating California’s policy to require opt-out HCV screening at all facilities would standardize programs across facilities, thus improving the identification of HCV infection persons without needing to “profile” individuals.

The successful implemented universal opt-out testing for HIV in California state prisons demonstrates the feasibility of expanded testing for HCV. In 2010 it led the USA, as the first state where all prison reception facilities tested every inmate for HIV, unless the individual declined (California Prison Health Care Services, 2010). In response, some state prison systems have implemented universal opt-out HCV testing. Opt-out HIV testing is popular among both prisoners and staff; between 60 and 85 percent of incarcerated individuals accept testing under opt-out models (Lucas, 2016; Basu *et al.*, 2005) and staff report that by having HIV testing integrated into larger intake protocols, opt-out testing requires less time than voluntary testing. Universal opt-out HIV testing resulted in high rates of linkage to care, retention in anti-retroviral therapy, and a significant reduction in viral loads among HIV-positive individuals receiving care during their prison sentence (Lucas, 2016). California’s successful implementation of opt-out HIV testing across facilities shows routine testing within correctional facilities is not only feasible but can help improve care and safety of correctional populations.

**Figure 1:**
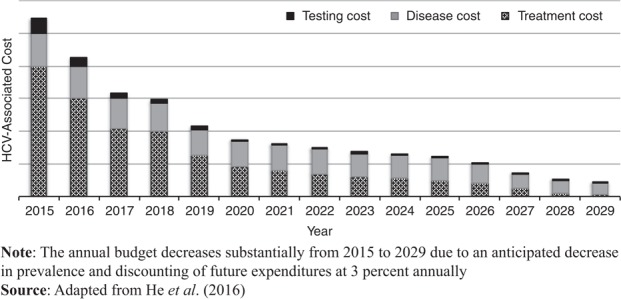
Budget needed to screen, treat, and manage HCV in prisons under universal opt-out policy (estimates based on a one-time test at entry)

Opt-out HCV screening has been adopted for other US populations. Center for Disease Control and Prevention (2012) recommended HCV screening for adults born between 1945 and 1965 in the general population (Centers for Disease Control and Prevention, 2012). While people born between 1945 and 1965 are more likely to than other adults to be infected with HCV, fewer than 0.2 percent of people in the general community test positive (Centers for Disease Control and Prevention, 2015). Universal opt-out HCV screening among prison populations is better at identifying HCV cases than general population testing (He *et al.*, 2016). Opt-out HCV screening translates to more individuals being diagnosed, and earlier – when paired with HCV treatment, opt-out HCV screening within prison is cost-effective and can have positive effects on HCV transmission within the general population. Figure 1 adapted from He *et al.* (2016) depicts a cost-savings example for implementing an opt-out policy at entry into all California state prisons. While the initial monetary investment is considerable for the resources for opt-out testing, most of which are attributable to the high price of DAA treatment for HCV, the cost drops significantly over time. Projections suggest opt-out testing, care, and treatment become a very small percentage of prison’s overall healthcare budget after 15 years due to a decrease in HCV prevalence in the facility and general community (He *et al.*, 2016; Kyckelhahn, 2012).

Of the 37 California state correctional facilities, 11 are also reception centers for inmates entering prison with sentences of two or more years. Currently only three reception centers provide opt-out HCV screening to all prisoners upon entry to their facilities. Implementing universal HCV screening across all California prison facilities would provide a consistent standard to identify HCV positive inmates. Equitable access to testing can improve budgetary resources for care. Additionally, the HCV test would become part of the streamlined medical intake protocol and therefore less stigmatized.

## Recommendations

1. Require all facilities within a correctional system to offer opt-out HCV screening upon entry, ensuring equitable access to testing for all individuals moving through correctional facilities. This recommendation additionally meets the US legal requirement for facilities to provide health care to be “consistent with acceptable community standards” (US Department of Justice, 1997).
2. Provide training for all medical intake staff on opt-out HCV screening and post-test disclosure counseling. Doing so will ensure a quality standard by which HCV screening is implemented. By packaging screening programs with test disclosure counseling, screening upon entry to correctional facilities will substantially decrease the number of individuals (estimated at 40-70 percent) who are unaware of their HCV status (Denniston *et al.*, 2012).
3. Couple testing policies with needed services to protect against HCV transmission in correctional facilities. The majority of prisons lack harm reduction services to prevent and educate individuals. Improved identification of persons infected with HCV will allow evidence-based budgetary decisions for facilities to package testing with prevention, education, and care services.

## Conclusion

As the California universal opt-out HIV testing example illustrates, requiring all correctional facilities to provide opt-out HCV screening upon entry ensures more accurate HCV detection, equitable access to HCV screening, and streamlined systems for more efficient work environments.

Universal opt-out screening offers several specific benefits. First, improved accuracy of recorded HCV rates in prisons. These data will inform evidence-based decision making for improved resource allocation by prison healthcare teams. Accurate estimates of the size of the HCV positive population can also help facilities assess the needs of their prison population. For facilities without a systematic screening program, the pool of infected individuals may be smaller than anticipated. By performing a one-time universal screening of all prisoners, the current HCV prevalence within a facility would be better understood. Second, early detection of HCV infections can improve HCV prevention and care. Equipping individuals with the knowledge of their HCV status upon release may help prepare parolees to access medical treatment and harm reduction programs in the community, potentially reducing transmission of HCV. Third, reduced stigma for individuals with or at risk for HCV, and subsequently reduce the risk of HCV transmission in prison and community populations. When people know they have HCV, they are less likely to engage in risky behaviors and are less likely to spread infection. Fourth, increase access to new HCV curative therapies. Delaying infection detection can delay or deny HCV care. Failure to treat may result in decompensated cirrhosis and hepatocellular carcinoma leading to high-intensity healthcare utilization, frequent hospitalizations and, potentially, liver transplantation. And fifth, offers a more cost-effective program, than voluntary or risk-based screening programs because HCV is treatable and curable. The advent of efficacious treatment with a high cure rate now makes wide-scale HCV screening an effective public health intervention.
